# Supplementary material for: Are carbon emissions trading and green financial instruments synergistic? -Comprehensive quantitative research based on content analysis
Source: PLoS One. 2024 Mar 7;19(3):e0298601. doi: 10.1371/journal.pone.0298601 (PMC10919601; doi:10.1371/journal.pone.0298601)
Supplement: S1 Data — (ZIP) [file pone.0298601.s001.zip › Data/地级市Z_F工作报告.zip!/╔┬╬≈/▒a╝a╩╨/└┤╫╘╣1⁄2╓┌║┼í╛┬φ┐╦╩2╛▌═°í┐_2016.docx]

各位代表：

现在，我代表市人民政府向大会作工作报告，请予审议，并请市政协委员和其他列席人员提出意见。

去年和“十二五”工作回顾

2015年，市政府认真贯彻中央和省、市委决策部署，牢牢把握“稳中求进、改革创新”的总基调，积极应对宏观经济趋紧、下行压力加大的形势，突出做大总量、做优质量两大要务，打破常规扩投资，精准施策稳增长，“双千”活动、PPP项目、渭河百里画廊建设、乡村旅游、夜间经济、治污降霾等一系列创新性工作成效显著。全年实现地区生产总值1788.59亿元，增长10.4%，高于全省2.4个百分点；财政总收入达到195.51亿元，增长3.9%；城乡居民人均可支配收入分别达到29475元、9511元，增长8.5%和9.5%，较好地完成了市十四届人大五次会议确定的目标任务，实现了“十二五”的圆满收官。

“十二五”期间，市政府坚持扭住发展不动摇，以人为本惠民生，解放思想，奋力拼搏，全市发展取得了令人瞩目的成就。

经济结构不断优化，综合实力迈上新台阶。全市经济总量大幅跃升，运行质量和效益稳步提高，三次产业结构比达到9.2:64.4:26.4 。地区生产总值年均增长12.7%，分别高于全国、全省4.9和1.6个百分点。人均生产总值达到7340美元,是2010年的1.86倍。实现地方财政收入84.47亿元，是2010年的2.18倍。非公经济、县域经济占地区生产总值的比重分别达到50.1%和48.5%，比2010年提高1.8和3.5个百分点。全市实现规模以上工业增加值838.73亿元,是2010年的1.9倍。外贸进出口总额五年累计达到42亿美元,是“十一五”期间的1.5倍。旅游业发展势头强劲，综合收入是2010年3.63倍。法门寺成功创建为国家5A级景区，实现了零的突破。农业园区带动特色产业规模扩张，苹果、猕猴桃面积分别达到121.9万亩、56.7万亩。

招商引资成效显著，项目建设取得新突破。全市招商引资累计落实到位资金 2417.87亿元，引进亿元以上大项目872 个、10亿元以上93个，吉利汽车、青岛啤酒、农夫山泉等知名企业落户我市。全社会固定资产投资累计达到8684.96亿元，是“十一五”期间的3.5倍。一批重大项目顺利推进，西宝客专、宝麟铁路、宝平高速、西宝高速、绛法高速改扩建工程建成通车，宝兰客专、宝汉高速开工建设；郭家河煤矿、150万吨甲醇一期、宝氮搬迁技改一期、通家新能源汽车等重点工业项目相继投产，开元商城、中国钛材交易中心、国家级眉县猕猴桃批发市场等商贸流通项目建成投用。

城镇体系更加完善，带动能力实现新提升。以做强高新区、重塑蔡家坡、建设新蟠龙为重点，大城市建设在规模扩张、功能完善上实现新突破。主城区面积达到106平方公里，比2010年增加15平方公里。实施了以经二路、金台大道为重点的旧城改造工程，城市面貌全面提升。建成石头河水库向市区及沿线城镇供水工程，全面完成了县区供水管网改造。五级城镇体系基本形成，64万农民进城落户，全市城镇化率预计达到49%，比2010年提高7.61个百分点。

人居环境持续改善，区域竞争形成新优势。实施绿色宝鸡“九大工程”，全市森林覆盖率达到55.26%，比“十一五”末提高1.48个百分点。治污降霾强力推进，去年市区空气质量二级及以上天数达到272天，连续两年位列关中各市之首。渭河综合治理工程主体竣工，新增河道水面100多万平方米，基本实现渭河干流全面截污。国家卫生县城实现全覆盖，美丽乡村建设持续推进，619个省级示范项目全面完成，生态文明创建工作走在全省前列。

重点改革稳步推进，经济发展呈现新活力。下放取消行政审批事项195项，市级行政审批事项总数减少35.6%，新增市场主体4.9万户，荣获中国驰名商标13件。市县政府机构改革、公务用车制度改革基本完成，农村土地确权登记颁证全面展开，岐山县、陇县农村产权交易中心建成投运。国有企业改革取得新突破，市属企业所有者权益增长72.2%。园区管委会体制改革稳妥推进，蔡家坡经开区、文管会和蟠龙新区管理体制进一步理顺，高新区成为西北地区唯一获批的国家创新型科技园区，凤翔、蟠龙新区和眉县科技工业园，分别被省政府批准为省级高新区、省级经开区。金融支持实体经济的能力不断提高，新增金融机构56家，农商行、“新三板”挂牌企业、创业投资基金、产业投资基金实现了从无到有。国家创新型试点城市建设成效显著，科技对经济增长的贡献率达到58%。

社会事业全面发展，人民生活再上新水平。全市新增财力的82.8%用于民生支出，民生十大工程累计完成投资103.3亿元。教育创强走在全省前列，建立了全学段、无缝隙教育资助体系。率先在全省推行公办医疗机构药品零差率销售，医改宝鸡模式、农村幸福院建设经验在全省推广，25万老年人喜领高龄补贴。建成文化产业示范基地14个，成为国家公共文化服务体系示范区。承办游泳、射击等国内赛事33次，成功创建全国全民健身示范城市。“大社保”宝鸡模式在全国推广，累计实现城镇新增就业27.5万人，农村劳动力转移就业年均100万人，荣获全国创业先进城市。建设保障性住房21.22万套。全面完成5910户农村“三无户”移民搬迁，在全省率先解决了农村特困户安全住房问题，累计实现50.6万农村贫困人口脱贫。公众安全感、群众满意度、信访维稳工作名列全省第一，被省政府命名为依法行政示范市。

同时，监察、审计、国土、统计、质监、安监、人防、物价、气象、档案、地震、地志、民族宗教、国家安全、妇女儿童、侨务外事、红十字会等工作也取得了新的成绩。

各位代表，回顾“十二五”，我们创新实干、精准发力，全市经济行稳致远，始终稳居全省第一方阵，为关天副中心城市建设提供了强有力的支撑；我们众志成城、攻坚克难，办成了一件件难事大事，建成全国文明城市，荣获中国十佳最干净城市、最中国十大美食城市称号，成功创建国家生态园林城市和全国幸福家庭示范市，举办了第27届世佛联大会、省第十五届运动会，宝鸡的美誉度和影响力大幅提升；我们集中财力、集中民智，着力解决好民生难题，基本实现了养老就业有保障、贫困家庭有住房、社区服务广覆盖、油路网络通到村的美好景象。这些成绩的取得，是在市委的正确领导下，市人大、市政协监督与支持的结果，是全市人民齐心协力、艰苦奋斗的结果。在此，我代表市政府，向人大代表、政协委员、各民主党派、各人民团体、社会各界人士，向驻宝企事业单位、人民解放军、武警驻宝部队全体官兵、公安干警，表示崇高的敬意和诚挚的感谢！

在充分肯定成绩的同时，我们应该看到，全市经济结构不尽合理，传统产业占比较高，非公经济和新兴产业发展滞后，第三产业总量不大、质量不优，保持中高速增长的动力不足；工业主导产业受体制制约比较明显，大项目仍然偏少，非税收入占财政收入比重偏大，整体经济实力还不够强；城乡二元结构明显，一些县域发展乏力，缺少新的增长点，脱贫攻坚任务繁重；干部的作风和能力与新形势要求仍有差距。对于这些问题，我们必须高度重视，努力加以解决。

“十三五”的思路目标和战略任务

“十三五”时期，是宝鸡全面建成小康社会的决胜期、追赶超越的关键期，也是融入“一带一路”战略、建设关天副中心城市的冲刺期。当前，国际经济疲弱复苏，发展环境日趋复杂，我国经济将进入“L”型的发展阶段，面临稳增长与调结构的双重压力，宏观形势对我市由间接影响变为直接传导。但是，应该看到，新一轮科技革命、产业转型升级给我们带来了新的发展机遇，省委、省政府也赋予了宝鸡更大的使命。同时，经过十多年的快速发展，宝鸡已经站在了新的历史起点上，具备了新常态下中高速发展的基础和能力。只要我们正确把握大局，顺势而为，不断增强发展意识、责任意识、忧患意识，善于捕捉新机遇，努力争创新优势，就一定能够再创宝鸡发展的新辉煌。

根据市委十一届九次全会的总体部署，“十三五”期间全市经济社会发展的基本思路是：全面贯彻党的十八大、十八届三中、四中、五中全会及习近平总书记系列重要讲话精神，坚持以“四个全面”战略为统领，以五大发展理念为指导，以追赶超越、群众满意为主线，突出投资增效、工业强基、旅游带动、县域突破、脱贫攻坚五大重点，加快打造关天副中心，建设最具幸福感城市，始终走在建设“三个陕西”前列，确保与全国全省同步够格全面建成小康社会。

主要预期目标是：到2020年，地区生产总值年均增长10%左右，达到3000亿元，人均生产总值超过1万美元；地方财政收入年均增长10%左右；城乡居民人均可支配收入年均分别增长10%和11%左右，努力达到全国平均水平；全社会固定资产投资年均增长13%；战略性新兴产业增加值占生产总值比重达到15%，研发经费投入强度达到3.5%；单位生产总值能耗累计下降16%，主要污染物排放总量、单位工业增加值用水量显著减少。

打造关天副中心，核心是实现“三大目标”：提升宝鸡工业优势，建设西部工业强市；打响炎帝故里、周秦文化、东方佛都、秦岭中央国家公园等文化旅游品牌，建设彰显华夏文明的文化名城；提高城乡基本公共服务水平，建设生态优美、人民生活富足安康的幸福家园。建设最具幸福感城市，关键是实现“四个同步提升”：以加快发展为第一要务，追赶全国、超越全省，实现经济发展与居民收入同步提升，增强群众富足感；以成功创建生态文明示范区为引领，让绿色尽染陈仓，让宝鸡的天更蓝、水更清，实现生态环境与城乡建设同步提升，增强群众舒适感；以打造全省最安全城市为目标，全面推进社会治理体系和治理能力现代化，实现民主法治与社会治理同步提升，增强群众安全感；以全面完成脱贫攻坚任务为头等大事，让全市人民共享改革发展成果，实现民生事业与文化建设同步提升，增强群众自豪感。

“十三五”时期，着力推进六大战略任务：

——着力推进产业转型升级，再造发展新势能。围绕建设创新型城市，以“智能化、绿色化、服务化、高端化”为方向，积极实施中国制造2025和“互联网+”战略，做大做强支柱产业，培育壮大新兴产业，改造提升传统产业，力争工业产值翻一番。把旅游业作为第三产业的重要引擎，坚持生活性服务业与生产性服务业并重，实现第三产业增加值900亿元。按照“做优特色、做大板块、做强龙头”的思路，推进畜牧、果品、蔬菜、粮食等四大主导产业转方式、调结构、提效益，力争农业总产值突破500亿元。重塑现代产业体系，让宝鸡的优势产业更优，特色产业更亮，产业实力更强。

——着力推进城乡一体发展，加速城镇群崛起。坚持科学规划与开发建设并举、管理提升与形象建设同步，完善基础设施，增强承载功能，彰显文化特色，吸引人口聚集，加快中心城区、城市副中心、县城、重点示范镇、特色小城镇、新型农村社区建设，到2020年，城镇化率达到60%，中心城区建成区面积达到143平方公里，让一个产业发达、特色鲜明、田园相间、生态宜居的城镇群呈现在关中西部。

——着力推进精准扶贫，坚决打赢脱贫攻坚战。以连片特困县和西部山区为重点，推动北部乔山、西部关山、南部秦岭三大重点区域全面攻坚脱贫。到 2017年全市33.1万农村贫困人口实现整体脱贫，2019年5个贫困县全部摘帽，贫困地区基本公共服务领域主要指标达到全市平均水平，不让一户家庭受困、不让一个群众受穷。

——着力推进重大设施建设，增强发展支撑能力。巩固提升交通枢纽地位，加快构建综合运输体系，形成“两横三纵七辐射”的铁路网、“十字加外环”的高速公路网、“两横两纵一环”的干线公路网，建成宝鸡机场，实现县县通高速，形成以市区为中心的快捷交通圈。加强重大水源工程建设，全面改善提升县城供水体系，农村群众饮用水全部达标。2018年实现全市管道天然气全覆盖。实施宽带宝鸡、云上宝鸡、数据宝鸡战略，加强网络安全，加快智慧城市建设，信息化总体水平进入全国先进行列。

——着力推进生态文明建设，巩固提升环境优势。精心打造“绿色宝鸡”生态品牌，构建城镇村庄风景如画、河流湿地清澈如镜、沿路沿渠林带如网、台塬坡面森林如屏的生态体系，率先在关中地区实现大地园林化。强力推进节能减排，大力发展循环经济，细化治污降霾举措，到2020年，森林覆盖率达到56%以上，建成二星级国家生态园林城市，实现国家园林县城全覆盖，保持空气质量关中最优、渭河水质流域最好、城乡环境全省最美。

——着力推进公共服务保障，持续提高群众幸福指数。坚持“保基本、兜底线、促公平、可持续”的原则，加大民生投入，提标扩面增项，创新服务机制，推进基本公共服务均等化。全面普及学前三年和高中阶段教育，逐步实现13年免费教育，推进中等职业教育免费，支持宝鸡文理学院建成宝鸡大学，在关天经济区率先基本实现教育现代化。加快建设健康宝鸡，到2020年，覆盖城乡居民的基本医疗卫生制度基本建立，城乡居民个人自付医药费用降至30%以下。

2016年的主要工作

今年是“十三五”开局之年。政府工作要以打造关天副中心、建设最具幸福感城市为目标，以稳增长、调结构为主基调，以改革创新为动力，以去产能、去库存、去杠杆、降成本、补短板五项工作为重点，以做强优势，推进有质量、有效益、可持续发展为基本要求，确保“十三五”稳健起步、良好开局。全市经济社会发展的主要预期目标是：生产总值增长10%左右，地方财政收入同口径增长10%左右，城镇登记失业率控制在4%以内，城乡居民人均可支配收入分别增长9%和10%左右，全社会固定资产投资增长16%，万元GDP能耗下降3.5%，主要污染物排放量完成省考目标。

重点做好七个方面的工作：

一、以项目建设为支撑，推动有效投资较快增长

扩大投资增量，优化投资结构，提高投资质量，切实发挥有效投资对经济增长的关键作用。

抓项目，促投资。按照“五个一”的要求，完善问责倒逼、定期协调和现场观摩制度，加快推进345个市级重点项目建设，确保完成年度投资606亿元。继续开展优化环境专项整治活动，严肃查处干扰项目建设和企业生产的不法行为，下大力气解决项目“落地难”问题，推动央企进陕、西洽会等签约项目实施，让投资者安心、放心、专心发展。

抓招商，促投资。继续实行专业小分队招商、主要领导定期招商机制，紧盯世界500强、民企100强和中省大型企业，有效开展产业链、点对点精准招商，确保全市招商引资到位资金增长15%以上。办好第二十五届中国西部商品交易会。坚持招商与招才并举、引资与引智并重, 全市引进3—5个投资过30亿元的大项目，高新区、蔡家坡经开区、蟠龙新区各引进2个以上过20亿元的产业项目，各县区至少引进1个过10亿元的项目，在项目策划、招大引强上实现再突破。

抓机遇，促投资。抢抓国家“一带一路”、新型城镇化、发展新兴产业等战略机遇，在交通基础设施、水利工程、产业升级、民生等领域，加大向上争取项目力度，争取中省资金不少于180亿元。超前谋划地下综合管廊、养老产业、城市停车场等补短板项目，争取国家专项建设债券。加快实施总投资120亿元的30个国家专项建设基金项目，确保国家支持政策落地生根。

抓平台，促投资。建立多层次资本市场，出台创新投融资体制的实施意见，设立政府投资引导母基金、产业发展专项基金，组建一批投融资运营公司，提升市县融资担保、土地储备运营、国资经营水平，为基础设施、产业发展、招商引资、公共服务等重点领域，提供更多的资金保障，年内社会融资规模达到170亿元。加大金融改革力度，推动金融产品创新，组建宝鸡农商银行，支持扶风建设浦发村镇银行，年内新增贷款120亿元以上。加快西凤酒股份首发上市，扶持一批中小企业挂牌，支持上市公司、“新三板”挂牌企业、优势企业通过资本市场融资。狠抓PPP项目建设，推动民间投资增长20%以上。

二、以破解结构性矛盾为关键，做大做强工业经济

把做优做强工业作为供给侧结构性改革的重中之重，充分发挥市场主导、企业主体、政府引导三个作用，制定出台加快工业强市建设的意见，实施工业“1553”行动计划，突出“五优化、五提升”，推动工业集群化发展。

优化结构，提升产业层次。立足存量，着眼增量，实施分类精准指导。优势支柱产业上档次、扩规模，加快推进吉利汽车产业基地、法士特轻卡变速器等项目建设，发展壮大汽车及零部件产业；引导钛材加工企业密切协作，延伸产业链条，打响“宝鸡·中国钛谷”品牌，发展壮大钛及钛合金产业；强力推进西凤酒城建设，确保青啤宝鸡工业园一期项目达产达效，推动乳品、饮料、面粉、方便面等农产品加工企业提质增效，发展壮大烟酒食品产业；加快煤炭资源开发利用，推进园子沟煤矿、招贤煤矿、北马坊二矿建设，抓好150万吨甲醇二期、麟游低热值煤发电等项目，发展壮大高端能源化工产业；支持宝石机械、宝石钢管等企业在中西亚、中东等地建设生产基地、设立销售机构，开拓国际市场，加快高端装备石油产业园二期、轨道交通科技产业园、高锰钢辙叉生产基地建设，发展壮大装备制造产业。战略新兴产业抓机遇、抢先机，推动宝成航空等军工企业与民品企业合作，重点发展无人机、旋翼机、通讯导航等产品，加快 7107厂航天产业基地建设，加速航空航天产业发展；推进宝华电动货车、龙源风电四期等项目建设，支持通家电动车完善生产线、开拓大市场，加速新能源及节能环保产业发展；加快蟠龙新区光通讯产业园、长岭科技工业园、烽火通信车、凌云密封铅蓄电池等项目建设，壮大渭滨区互联网产业园，加速电子信息及光通讯产业发展；支持辰济药业、华西制药、秦明电子、西北机器等制药企业和医疗设备企业扩大产能，建设紫光医药科技产业园，加速生物医药与健康设备产业发展；加大工业智能机器人关键件研发力度，积极推进秦川机床机器人关节项目，加速智能制造产业发展。传统特色产业抓提升、促转型，鼓励水泥等建材企业“走出去”，实现产能转化、产品升级，加快岐山新型建材工业园、千阳建筑陶瓷环保产业园转型升级，提升建材产业发展水平；推动3户市属纺织企业整合重组，推进岐山普宁服装产业园建设，引导纺织、服装企业开展技术改造、促进产品升级，提升纺织服装产业发展水平；推动铅锌矿产资源的重组整合，组建采、选、冶、加一条龙的大型矿业集团，提升有色冶金产业发展水平。加大国企改革力度，引导企业实施兼并重组、债务重组和破产清算，淘汰“僵尸企业”、高污染企业、产能过剩领域无竞争力企业，实现市场出清。力争规模以上工业增加值增长10.8%。

优化创新，提升增长动力。围绕主导产业，整合科技资源和项目，加大重点企业扶持力度。围绕新技术、新产品，支持新能源汽车、油电两用发动机等项目建设。围绕创新能力体系建设，争取建立钛产业全国创新中心，积极与清华大学苏州汽车产业园合作建设汽车技术研发平台，不断加强与省内外高校、院所产学研联合，建立与西安、北京、上海及国外的协同创新平台，把宝鸡建成科技成果转移转化的承载基地。围绕传统产业转型升级，深入实施“两化”深度融合6个专项行动，完成宝石钢管、西凤酒、中铁电气化器材厂3户企业国家级“两化融合贯标”工作，推进20户省市级两化融合试点项目建设。围绕产业迈向中高端，建立引进培育高层次创新创业人才激励机制。

优化载体，提升园区配套。推进产城融合，把工业园区纳入城镇发展总体规划，科学合理布局，适度控制规模，集约利用土地。加快水、电、气、路、讯等基础设施建设，完善学校、医院、金融、商贸等服务设施，提升园区承载能力。加大标准化厂房建设力度，年内建成标准化厂房3万平方米，实现县县有预备的标准化厂房，为中小企业入园发展创造条件。探索建立市场化运作、多元化投入机制，筹措更多园区建设资金。创新园区体制机制，引进高层次管理人才。加强园区投融资管理，增强自我发展能力。支持重点园区建设，出台进一步加快高新区发展的意见。

优化主体，提升发展活力。继续深化商事制度改革，吸引民资、激发民智，促进大众创业、万众创新。支持非公企业参与国有企业改制重组，推进国资与民资融合、军民融合，拓展非公经济“成长空间”。积极实施百户科技型中小企业培育工程，开展扶助双创型中小微企业专项行动计划，落实财政扶持、税收优惠、用地保障等政策措施，多渠道解决中小企业融资难题，助推非公企业“破茧化蝶”，年内培育规模以上工业企业30户。

优化服务，提升要素保障。围绕支柱产业和新兴产业，组建专门机构、抽调专门人员、学习专门知识、制定专门计划、加强专业化指导、推进专业化招商，切实提高服务工业经济的能力和水平。继续搞好“双千”活动，建立共性问题联合处理机制，搭建反映问题网络平台，定期开展银企对接、项目对接、政策进企业等活动，形成服务企业的长效机制。进一步推动企业减负工作，认真落实中省各项惠企政策，打好“降本增效”组合拳，加快剥离企业办社会职能，解决历史遗留问题，减轻企业税费负担，扩大直供电试点范围，让更多企业享受政策“红利”。

三、以文化旅游为龙头，引领第三产业发展

做宽领域、做大影响、做旺人气，促进第三产业加快发展。第三产业增加值达到518亿元，增长9%。

围绕大众需求，推动文化旅游产业上水平。观光游、休闲游、度假游融合发展。依托山水资源，发展旅游+生态，突出宝鸡“生态”卖点，加快推进关山草原、黄柏塬、大水川等景区和渭河百里画廊建设，推动太白山景区5A级创建。启动建设天台山大景区，打造都市生态休闲核心区。依托人文资源，发展旅游+文化，推出一批具有宝鸡地方特色的影视剧、实景演艺等文化精品力作，满足游客文化需求，加快法门寺景区全面提升，开工建设扶风大美佛汤城，推进西周文化景区等建设。依托民俗资源，发展旅游+休闲，将休闲观光旅游与感知宝鸡风情、体验西府民俗、品味农家生活有机结合，加快休闲民俗重点村镇建设，实现批量向游客开放。依托最中国美食城市，发展旅游+美食，深度挖掘饮食文化内涵，制定美食街区规划，评定特色美食示范店，推进岐山臊子面产业园项目，让游客尽享宝鸡物产之美，感受宝鸡味道。依托重要赛事，发展旅游+体育，大力推进“赛事兴旅”，吸引游泳跳水、射击射箭、田径球类、武术散打、太极拳等国内外重大赛事来宝鸡举办，深度开发户外健身休闲项目，丰富旅游产品结构，推进太白县高山滑雪场建设。创新体制机制，推动旅游投资、建设与管理市场化步伐。加大精品景点和旅游基础设施建设力度，设计黄金线路，带动旅游业发展。开发体验性、互动性旅游产品，精心办好2016宝鸡文化旅游节和太白山·韩国文化旅游年。加强旅游标识体系建设，开展多元化、立体式的宣传营销活动，让游客来得了、留得住、有的看、有的吃、有的买，打响宝鸡旅游品牌。全年接待游客突破6000万人次，实现旅游综合收入410 亿元。

围绕消费需求，推动商贸流通业扩规模。健全商贸流通体系，加快夜间经济八大示范点特色化发展，推进东岭国金中心建设，建成银泰购物中心、千禧时代商业街等商贸设施，新发展30个服务业聚集区，打造15分钟便民消费圈。实施肉菜流通追溯体系、社区蔬菜流通网络、放心早餐等商贸惠民工程。支持眉县猕猴桃等外贸基地建设，搭建全市外派劳务平台，组织企业走出去，开展产品展销和经贸合作活动，积极发展跨境电子商务，开拓国际市场，促进外贸优进优出，全市进出口总值突破9亿美元，增速保持全省前列。推进阳平铁路物流中心建设，力争宝鸡综合保税区开工。全年实现社会消费品零售总额686.5亿元，增长12%。

围绕市场需求，推动新兴业态快成长。大力发展生产性服务业，推动研发、设计、物流、会计、运维等行业向专业化和价值链高端延伸。大力发展生活性服务业，积极发展医疗保健、健康养生、文体休闲、养老、家政等新型消费，满足群众多样化、个性化消费需求。大力发展电子商务产业，加强与阿里巴巴、京东、苏宁易购等互联网企业合作，深入推进“百镇千企万人”电商创业行动，抓好省级电子商务示范县、示范园区创建，实现农村电商全覆盖，让宝鸡的更多产品网走四方。

四、以精准扶贫为重点，推动农村全面发展

用发展新理念破解“三农”新难题，坚持强农惠农富农政策不减弱，以特色产业带动县域经济发展，推进农业强、农村美、农民富。

转变方式，提高农业效益。推动传统农业向优质高效农业转变，加快发展现代农业。调整农业内部结构，提升粮食综合产能，高标准改造奶牛规模养殖场50个，扶持肉牛肉羊规模化养殖场、家庭牧场82个，新发展苹果10万亩、核桃5万亩、猕猴桃3万亩、花椒1万亩，支持蔬菜产区扩大规模。转变农业经营方式，推进原料生产、加工物流、市场营销等一二三产融合发展。加快土地流转步伐，积极培育专业大户、家庭农场、农民合作社、龙头企业等新型农业主体，支持新型农业服务机构开展代耕代种、联耕联种、土地托管等专业化规模化服务。打造农业特色品牌，建设2个省级、15个市级现代农业园区，力争太白县成为国家级现代农业示范区，打响眉县猕猴桃、凤县花椒、陇县配方羊奶粉、太白山无公害蔬菜等品牌，抓紧注册宝鸡苹果公用商标，建立蜂产业科研机构和检测中心，加快蜂产品开发步伐。推进农村产权制度改革，全面完成农村土地确权登记颁证，推进土地经营权抵押、担保试点，建立健全四级土地流转服务体系，一半以上的县区建成农村产权交易中心。搞好第三次农业普查。

分类指导，实施精准扶贫。带着责任、带着感情，真扶贫、扶真贫，打好新一轮扶贫攻坚战。发展生产脱贫一批，立足当地资源，制定贫困地区特色产业发展规划，宜农则农，宜牧则牧，宜游则游，通过扶持发展特色产业，实现就地脱贫。易地搬迁脱贫一批，本着靠近城镇、靠近园区、靠近中心村的原则，坚持“政府主导、规划引领、整合资源、多方筹资、集中安置”的思路，对生产生活条件较差的贫困群众，实施移民搬迁5400户、2万人，搞好基础设施配套，完善公共服务，让贫困群众搬得出、稳得住、能致富。生态补偿脱贫一批，把生态环境保护与扶贫攻坚一体谋划，依托天然林保护、防护林工程、林业产业，加大退耕还林还草力度，提高政府补贴，增加群众收入。发展教育脱贫一批，把职教扶贫作为重要抓手，深入推进职业教育改革，整合各级各类培训资源、培训资金，加强对贫困群众的技能培训。开展免费职业技能鉴定，对赴外省务工人员给予一定补贴，支持企业优先吸纳贫困人口就业，让有能力的贫困群众都有一技之长，确保每个有条件的贫困家庭至少有一人打工就业，实现转移就业脱贫。全面落实教育扶贫各项优惠政策，坚决斩断贫困代际传递。社会保障兜底一批，实行农村低保标准与扶贫标准“两线合一”，加大医疗救助力度，对无劳动能力的贫困人口，由政府全额出资参加新农合，所有贫困县实现门诊统筹全覆盖，不让一个贫困群众掉队。年内，全市完成15.4万人脱贫，金台区、渭滨区实现贫困人口全部脱贫。

夯实基础，增强发展后劲。健全投入长效机制，建好、管好、护好、运营好农村基础设施。大规模推进农田水利建设，深入实施大中型灌区续建配套和节水改造，发展节水灌溉10万亩，新修改造基本农田4万亩。加快重大水利工程建设，实施千河防洪治理项目，建设石沟、崔木、长益庙水库，提升抗旱应急保障能力。抓好县乡公路和危桥改造项目，持续改善农村出行条件。

五、以新型城镇化为方向，提升规划建设管理水平

坚持以人为本，尊重城市发展规律，完善科学治理体系，不断提升城市的吸引力、承载力和竞争力。

高起点规划，彰显城市特色。制定出台加强城市工作的意见，按照“一主三副一带两翼”市域空间布局，完善重点地段控制性详规，推进“多规合一”。把独特风格作为宝鸡城市的灵魂与软实力，开展建筑风格、城市色彩和城市功能完善的规划研究，建设综合性城市数据库，加强对历史街区、建筑的有机保护，启动老街区特色化改造。严格执行规划，收紧规划管理权，确保城市规划红线不能碰，切实形成城市风貌的整体性和文脉的延续性，不断提升市民对城市的归属感、认同感、自豪感。

高标准建设，增强城市功能。树立经营城市理念，多渠道筹措建设资金。继续做强高新区，加大南客站片区、科技新城、陆港新区建设力度，打造西部创新之城；提速建设新蟠龙，高标准实施新区配套设施项目，建设科技生态示范区；加快重塑蔡家坡，建设汽车产业聚集区、幸福美丽小城市。推进海绵城市和地下综合管廊建设，加大地下空间建设利用，提高城市现代化水平。坚持完善功能与提升品质并举，开展城市“双修”工程，抓好年度城建项目，继续推进新东岭综合体等旧城和城中村改造，加强便民市场建设，进一步抓好城市亮化工程；完善城市路网，加快城市轻轨规划建设工作，加强渭河南北两岸交通联系，建设植物园、联盟路、阳平渭河大桥及宝烟什字立交桥，打通“断头路”。加快县城、重点镇和统筹城乡示范区建设，加大项目资金倾斜力度，全面实施县城提升改造工程，支持凤翔、陇县副中心城市提升品质、率先发展，支持凤县、太白县按照5A级景区规划建设、突破发展，支持其余县城、省级重点示范镇和文化旅游街区突出特色、加快发展，打造精品魅力城镇。全面落实信贷、税收新政，推进农业转移人口市民化。加快构建便捷交通网络，推进宝兰客专、宝汉高速、连霍高速宝鸡过境线、绛汤高速建设，抓好12个国省道改扩建和县城过境线建设项目，做好合凤高速、关中环线眉县汤峪至岐山凤鸣段等项目前期工作，推动宝鸡机场项目取得实质性进展。

高水平管理，提升城市品质。开展国家环保模范城市、国家生态园林城市、国家卫生城市、全国文明城市“四提升行动”。理机制，推进城市管理大部门制改革，整合市政公用、物业管理、市容环卫、园林绿化、城市管理等相关执法职能，依法构建权责清晰、服务为先、管理优化、执法规范的城市管理体制。补短板，围绕群众关心的交通拥堵、管理粗放、应急滞后等问题，下硬茬、出实招，有效缓解“城市病”。攻难关，大力推广垃圾分类试点和清洁收运，加快城市垃圾无害化处置方式转变。保重点，推进城市智能化、精细化管理，深入开展市容市貌整治，深化背街小巷、城乡结合部、农贸市场等重点区域治理。强化协调协同，推动政府、社会、市民同心同向行动，实现城市管理共治共管、共建共享。

六、以生态建设为抓手，优化人居发展环境

坚持生态优先、绿色发展，以系统化思维推进环境治理与生态保护，为持续发展保本钱、攒后劲。

大规模造林，营造一域绿色。深入实施绿色宝鸡“九大工程”，全面推进城市森林化、村庄林荫化、河流湿地生态景观化等“八化”建设。加快实施万亩森林围市，打造城在林中的宜居宜业环境。突出抓好主城区、县城和重点镇的绿化美化，深入推进生态园林县城、园林镇、园林单位、园林小区创建活动。建设围村片林，抓好村庄和道路、河流周边绿化美化。多栽植高大乔木树种，绿树成林成片，让群众在绿荫下享受生活。全年完成绿化45万亩、植树4000万株。

下功夫降霾，保卫一片蓝天。顺应群众对清新空气的期待，坚持源头严防、过程严管、质量严考、后果严惩，持续开展扬尘治理、清洁能源、工业减排、网格监管、监测考核、环境执法等六大行动，完成市区10个集贸市场清洁化能源改造，治理1万户沿街小火炉和100个工业项目，完成大唐热电、宝二电公司火电机组超低排放改造工程，实现大气污染治理“六个清零”。积极推进燃煤大户和能源化工企业的煤改气、煤改电工作，提高天然气和集中供热普及率，推广余热发电、地源热泵等技术，通过能源结构调整，从根本上减少污染排放，让群众看到更多的蓝天白云。

铁手腕治污，守护一方净土。巩固提升渭河治理成果，确保生态基流，把渭河打造成为宝鸡的中央公园。深入开展渭河治污，全面实施沿渭污水处理厂提标改造，建设沿渭城镇截污管网，使沿河单位、农村污水得到全面处理。整治渭河干流及支流排污口，确保直排渭河污水全截污、全收集、全处理。建成蟠龙桥到卧龙寺龙丰段生态湿地，实施千渭之会景观提升工程，抓好眉县龙源、扶风七星河等湿地公园建设，全市所有污水处理厂出水口建成人工湿地，进一步净化水质、美化环境。加强农村面源污染治理，严控规模养殖场超标排放。

全方位整治，描绘一幅美景。以“清洁家园、清洁田园、清洁水源、清洁能源”为目标，整合环保、住建、农业等部门力量，实行向上争取资金与财政兜底相结合，对尚未整治的630个村分类施治，全面完成40个村省级集中连片整治示范项目，推动农村环境综合整治全覆盖。积极破解难题，建立农村生活垃圾、污水处理长效机制。完善文体设施，加快村史馆建设，进一步丰富美丽乡村建设内涵。推进燃气进村及改炕、改灶试点。积极开展生态文明示范区“四级联创”。

七、以改善民生为根本，保持社会和谐稳定

坚持把群众满意作为衡量标尺，推进民生保障与社会事业同步提升，让全市人民的生活一年比一年更美好。

真情富民，让创业就业有门路。加快创业孵化基地建设，实施青年创业计划、大学生创业引领计划、农民工返乡创业计划。大力开展就业援助活动，帮助就业困难人员、失业人员实现就业，做好“去产能”分流人员就业工作。按照“失地不失业、减地不减收、一产转三产、农民变市民、收入有渠道、养老有保障”的原则，切实做好被征地农民安置就业工作。实现城镇新增就业4.5万人，农村劳动力转移就业85万人以上。

真情惠民，让教育医疗更优质。组建和深入推进城乡教育联盟，推动教育现代化先进县创建，在全省率先实现义务教育发展基本均衡县、“双高双普”县全覆盖。大力实施职业教育“1256”工程，为产业发展和群众增收提供有力支撑。深入推进公立医院改革，不断降低医疗费用，加快实施分级诊疗，方便群众就医。继续为基层培养全科医生，推动优质医疗资源下沉。全面实施两孩政策，促进人口长期均衡发展。开展健康城市建设，提高健康服务保障水平。

真情为民，让社保服务更方便。持续完善“大社保”模式，积极推行社会保障业务“一站式”服务、城乡居民基本养老保险“五个不出村”便民工作机制，推进“五险合一”统缴统管，开展全民参保登记计划试点，整合城乡居民基本医疗保险制度。推进社会保障卡建设工程，累计发卡220万张。加快34个社区服务中心项目建设，县城公益性公墓实现全覆盖。

真情暖民，让文化生活更多彩。加快建设市文化艺术中心等文化设施，抓好“话剧月月演、秦腔周周唱、电影假日放”等多种形式的文化惠民项目。力推大剧大作的录制，把《梁生宝买种记》推向全国巡演。引导和支持民间资本投资文化产业，建设产业孵化基地，启动眉县关学学院建设。完善城乡群众健身场地和设施，推进市县标准足球场建设，建成渭河健身长廊工程。

真情安民，让社会发展更和谐。全面启动“七五”普法，深化平安宝鸡建设，健全立体化治安防控体系，依法打击非法集资、传销等涉众型违法犯罪活动。继续深化信访制度改革，有效预防和化解社会矛盾。做好新形势下民族工作，依法管理宗教事务。创建成为国家食品安全城市，确保群众饮食用药安全。落实安全生产责任，强化源头管理，推进“智慧宝鸡·安全生产”系统建设，有效预防和遏制安全生产事故的发生。

各位代表，群众的幸福感就是政府工作的成就感。2016年，市政府将着力为全市人民办好十件实事：一是投资4800万元，新建农村幸福院483个、社区日间照料中心和养老服务站8个、县级社会福利中心1个。二是投资1.07亿元，实施50处农村供水巩固提升工程、7个抗旱应急水源工程，解决10万农村人口安全饮水问题。三是投资1.14亿元，建设26个省级、43个市级残疾人扶贫、康复、就业、托养、培训示范基地，帮助2.2万名残疾人精准脱贫，让10万残疾人从中受益。四是投资3000万元，实施技能培训就业惠民工程，实现初高中毕业返乡青年免费培训全覆盖。五是投资2亿元，启动农村公路进村“最后一公里”建设三年行动计划。六是按照市场化运营模式投资6亿元，在市区新增停车位6000至8000个，缓解“停车难”问题。七是投资7.5亿元，推进城市光网入户、农村光网进村工程，实现免费WiFi基本覆盖全市主要广场和旅游景区等公共区域。八是投资3.98亿元，实施县城以上城区中小学校建设、第二期学前教育三年行动计划、普通高中改造等项目278个。九是投资130亿元，完成2.03万户棚户区、城市危房改造。十是投资1亿元，对老旧小区进行改造提升，让居民生活更方便、更舒心。

切实加强政府自身建设

立足新起点，适应新常态，面对新期待，我们必须以更加高效的工作促进发展，以更加务实的作风服务群众，切实担当起推动宝鸡追赶超越的使命和责任。

加强学习研究，增强履职能力。知识是生产力，也是执行力。面对新形势，全体政府组成人员要更加注重学习理论、学习业务、学习先进，以政府工作人员知识结构、能力结构的优化，应对发展方式的转变。完善市政府集体学习制度，建立政府智库，定期邀请专家学者，开展针对性学习研究。坚持用新思想、新理念谋划推动工作，以创新举措应对复杂局面，不断提高科学决策能力和驾驭全局能力。

强化管理服务，提高政府效能。继续推进简政放权，加快建设智慧城市，启动“两云五通一中心”工程，构建网上政府，推进管理服务智能化。深化财政预算改革，加强财政绩效管理。在各级政府及工作部门开展“追赶超越、争先进位”活动，切实提高干部的“精气神”。坚持重心下移，大力推行“一线工作法”和“现场办公制”。实行重点工作与重点项目台账式管理，问事必问人、问人必问责、问责必问效。建立“容错机制”，调动和保护各级干部干事创业的积极性，确保各项工作高效落实。

严格依法行政，自觉接受监督。加强法治政府建设，坚持法定职责必须为，法无授权不可为，讲程序、讲规矩，依法、依规、依纪、依制度办事。进一步理顺行政执法体制，着力解决权责交叉、多头执法问题。深入推进公务人员尊法、学法、守法、用法，提高依法行政水平。自觉接受人大依法监督和工作监督，主动接受政协民主监督，认真听取民主党派、工商联、无党派人士和各人民团体的意见。继续开展“百姓问政”“万名群众评议百名科长”以及政风行风测评活动，积极接受舆论监督和社会监督，确保行政权力公开、透明运行。

坚持从严治政，树立良好政风。始终把党的政治纪律和政治规矩挺在前面，全面落实党风廉政建设责任制，严格执行“一岗双责”制度，从源头上预防腐败。进一步巩固提升“三严三实”专题教育成果，严格落实中央“八项规定”精神和国务院“约法三章”要求，驰而不息纠正“四风”，坚决整治和查处侵害群众利益的不正之风和腐败问题。从严控制行政成本，确保“三公”经费只减不增。强化政府内部制约与监督，实现审计工作全覆盖。

各位代表，新的起点描绘新的蓝图，新的一年孕育新的希望。让我们在市委的坚强领导下，以更加坚定的信心，更加昂扬的斗志，更加饱满的干劲，团结拼搏，锐意进取，努力实现“十三五”良好开局，为加快打造关天副中心、建设最具幸福感城市做出新的更大贡献！
